# Blast shockwaves propagate Ca^2+^ activity via purinergic astrocyte networks in human central nervous system cells

**DOI:** 10.1038/srep25713

**Published:** 2016-05-10

**Authors:** Rea Ravin, Paul S. Blank, Brad Busse, Nitay Ravin, Shaleen Vira, Ludmila Bezrukov, Hang Waters, Hugo Guerrero-Cazares, Alfredo Quinones-Hinojosa, Philip R. Lee, R. Douglas Fields, Sergey M. Bezrukov, Joshua Zimmerberg

**Affiliations:** 1 Section on Integrative Biophysics, Eunice Kennedy Shriver National Institute of Child Health and Human Development, National Institutes of Health, Bethesda, MD 20892-1855, USA; 2 Celoptics Inc., Rockville, MD 20852, USA; 3 Department of Neurosurgery, Johns Hopkins University, Baltimore, MD 21287, USA; 4 Section on Nervous System Development and Plasticity, Eunice Kennedy Shriver National Institute of Child Health and Human Development, National Institutes of Health, Bethesda, MD 20892-3713, USA; 5 Section on Molecular Transport, Eunice Kennedy Shriver National Institute of Child Health and Human Development, National Institutes of Health, Bethesda, MD 20892-0924, USA

## Abstract

In a recent study of the pathophysiology of mild, blast-induced traumatic brain injury (bTBI) the exposure of dissociated, central nervous system (CNS) cells to simulated blast resulted in propagating waves of elevated intracellular Ca^2+^. Here we show, in dissociated human CNS cultures, that these calcium waves primarily propagate through astrocyte-dependent, purinergic signaling pathways that are blocked by P2 antagonists. Human, compared to rat, astrocytes had an increased calcium response and prolonged calcium wave propagation kinetics, suggesting that in our model system rat CNS cells are less responsive to simulated blast. Furthermore, in response to simulated blast, human CNS cells have increased expressions of a reactive astrocyte marker, glial fibrillary acidic protein (GFAP) and a protease, matrix metallopeptidase 9 (MMP-9). The conjoint increased expression of GFAP and MMP-9 and a purinergic ATP (P2) receptor antagonist reduction in calcium response identifies both potential mechanisms for sustained changes in brain function following primary bTBI and therapeutic strategies targeting abnormal astrocyte activity.

Blast-induced traumatic brain injury (bTBI) continues to be a worldwide health problem. bTBI can be complex, resulting from one or more physical phases of the blast phenomenon. Even those experiencing low-level blast explosions, such as those produced by explosives used to breach fortifications, can develop neurocognitive symptoms without evidence of neurotrauma^1^. The cellular mechanisms of this phenomenon are unknown. The primary phase of bTBI, characterized by organ-shockwave interaction, is unique to blast exposure^2^. Understanding the mechanisms and pathology arising from the primary phase of bTBI is limited^3^,^4^,^5^,^6^, in part, because of the limited availability of *in vitro* models simulating the blast shockwave. Therefore, it is critical to develop experimental methods to study the primary phase of bTBI.

To better study the primary phase of bTBI, we developed a pneumatic device that simulates an explosive blast by producing pressure transients similar to those observed in a free field explosion and is compatible with real-time fluorescence microscopy of cultured cells; this device can produce blast-like pressure transients with and without accompanying shear forces^7^,^8^. Using Ca^2+^ ion-selective fluorescent indicators, changes in intracellular free calcium following simulated blast were detected. We previously showed that a) cultured human brain cells are indifferent to transient shockwave pressures known to cause mild bTBI, b) when sufficient shear forces are simultaneously induced with the shockwave pressure, central nervous system (CNS) cells respond with increased intracellular Ca^2+^ that propagates from cell to cell; and c) cell survival is unaffected 20 hours after shockwave exposure^7^. In this study we determine the cell type responsible for the waves of increased intracellular free Ca^2+^.

Astrocytes respond rapidly to traumatic brain injury, having both beneficial and deleterious effects in a wide range of pathological conditions. Under normal conditions, astrocytes also have important roles in integrating information and feedback modulation exists between astrocytes and neurons^9^,^10^. In response to mechanical strain, cell swelling, and cellular trauma, intercellular calcium waves can spread between astrocytes through gap junction mediated 1,4,5-trisphosphate (IP3) diffusion and by purinergic signaling in response to ATP released from cells. Astrocyte ATP release activates purinergic ionotropic subclass X (P2X), and purinergic metabotropic subclass Y (P2Y) receptors on other cells^11^,^12^ causing inter-cellular calcium waves among astrocytes. Astrocytes respond to secondary and tertiary phase central nervous system (CNS) traumas by altering their morphology and gene expression^13^. This “reactive” state is characterized by increased glial fibrillary acidic protein (GFAP) expression^14^,^15^,^16^. Reactive astrogliosis is postulated to have both beneficial and detrimental effects^16^,^17^.

We show that simulated blast primarily affects calcium signaling in human astrocytes producing calcium waves that propagate via purinergic signaling. Dissociated human CNS cortex cells, gestational weeks 19–21, are more responsive than dissociated rat CNS cortex, embryonic day 18. Two genes, astrocyte GFAP and matrix metallopeptidase 9 (MMP-9), have increased expression in human cell cultures and may be involved in longer-term brain effects associated with mild bTBI.

## Results

### Calcium propagation in dissociated CNS culture

**Figure 1:**
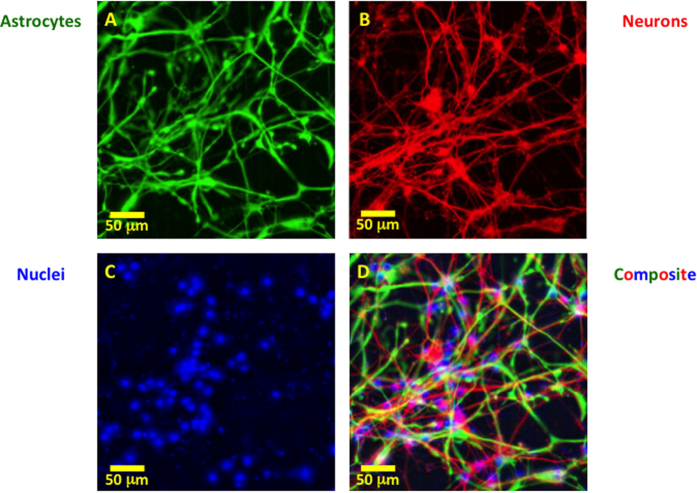
Immunostaining of dissociated human fetal CNS culture (21 Days in culture) labeled with astrocyte marker, GFAP (**A**), neuronal marker TUJ1 and MAP2 (**B**), nuclei marker Hoechst (**C**), and the composite overlay (**D**). Scale bar, 50 μm.

**Figure 2:**
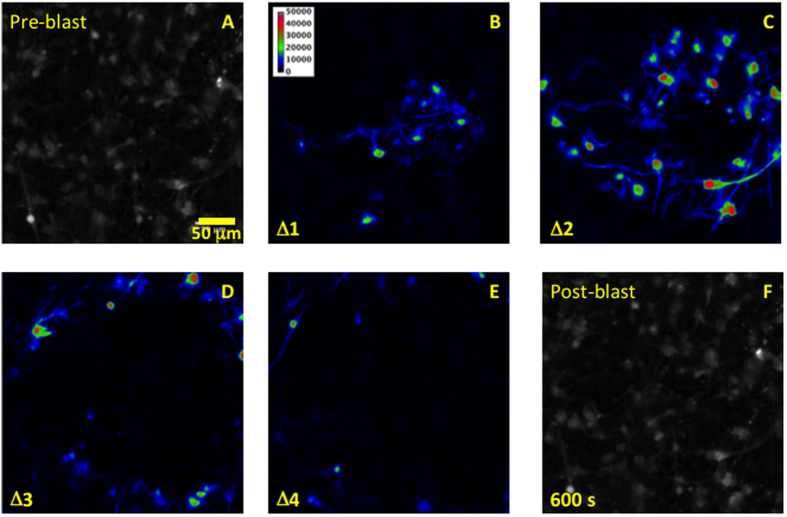
Calcium propagated response to blast shock wave. (**A**) Fluo-4 fluorescence image of the observation field prior to blast. (**B**–**E**) Pseudo-color consecutive differences between images representing the changes in free calcium concentration over the first 5 seconds following simulated blast. (**F**) The fluorescence image of the observation field at the end of the experiment. No loss of indicator from cells, due to acute damage, was observed. Scale bar, 50 μm.

Our dissociated human CNS cultures consist primarily of neurons and astrocytes (Fig. 1). In response to a blast-like shock wave that concomitantly causes shear forces, one or more propagating waves of increased intracellular free Ca^2+^ are observed^7^,^8^. Usually, the calcium waves propagate into the observation field, resulting in complex patterns due to multiple initiation sites within the well, often outside the field of observation. On occasion initiation of an outward, radially propagating wave of increased cytoplasmic free Ca^2+^ occurred within the observation field (Fig. 2 and Movie M1).

**Figure 3:**
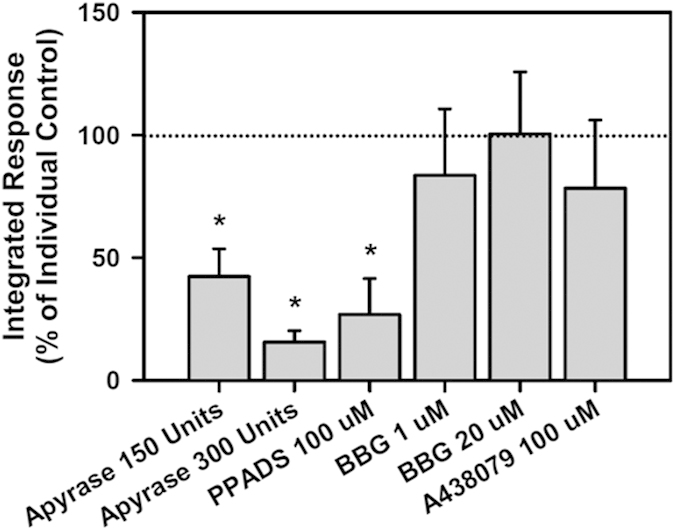
Calcium response to laser wounding propagates via purinergic signaling. The calcium response significantly decreased in a dose-dependent manner following enzymatic degradation of ATP and ADP by apyrase (n = 4, 4, and 5 for 0, 150 and 300 Units apyrase, p = 0.002 single factor ANOVA). Comparable to apyrase, the non-specific purinergic blocker, PPADS significantly blocked the integrated response (n = 4 and 4 for 0 and 100 μM PPADS, normalized reduction 0.27 (0.29) (mean (SD)) while the P2X7 specific blockers BBG and A438079 were without effect (n = 4, 4, and 4 for 0, 1 and 20 μM BBG; p = 0.76 single factor ANOVA and n = 4 and 3 for 0 and 100 μM A430789, normalized reduction 0.78 (0.55) (mean (SD)). The dotted line at 100% represents the individual controls associated with each experiment.

To investigate in this culture system the propagation of calcium activity from a defined initiation site and to investigate cellular mechanisms involved in intracellular free calcium wave propagation, laser wounding was used to localize the initiating site within the observation field. Laser wounding results in propagating waves of increased cytoplasmic free Ca^2+^ comparable to those observed using simulated blast. In principle, injury can occur in neurons or astrocytes through direct effects on each cell type. Blocking neuronal activity using TTX (1 μM; Sigma-Aldrich) alone or TTX (1 μM), (2*R*)-amino-5-phosphonopentanoate, (APV) (50 μM; Sigma-Aldrich), and 2,3-dihydroxy-6-nitro-7-sulfamoyl-benzo[f]quinoxaline-2,3-dione (NBQX) (50 μM; Sigma-Aldrich) to block excitability, NMDA and non-NMDA glutamate receptors, respectively, had no significant effect on the calcium response (integrated Δ F(t)/F_0_ time course, n = 4 for each condition, p = 0.19, single factor ANOVA). Since astrocyte calcium signaling in response to mechanical strain or cell swelling is known to occur through purinergic receptors, we tested whether the observed propagation of increased cytoplasmic free Ca^2+^ in this preparation is a result of purinergic activity. Cells were laser wounded in the presence of apyrase (Sigma-Aldrich), an enzyme that rapidly degrades extracellular ATP. A significant dose-dependent reduction in the calcium response was observed with increasing concentrations of apyrase (Fig. 3; n = 4, 4, and 5 for 0, 150 and 300 Units apyrase, p = 0.002 single factor ANOVA). The non-specific antagonist pyridoxalphosphate-6-azophenyl-2′,4′-disulfonic acid (PPADS) (Tocris Bioscience) significantly blocked the calcium response (Fig. 3; n = 4 and 4 for 0 and 100 μM PPADS, normalized reduction 0.27 (0.29) (mean (SD)). However, the P2X7 specific antagonists, Brilliant Blue G (BBG) (Sigma-Aldrich) and A438079 (Tocris Bioscience), did not significantly alter the calcium response (Fig. 3; n = 4, 4, and 4 for 0, 1 and 20 μM BBG; p = 0.76 single factor ANOVA and n = 4 and 3 for 0 and 100 μM A430789, normalized reduction 0.78 (0.55) (mean (SD)). Our observed neuronal and purinergic blocker dependencies support the hypothesis that astrocytes are involved in the response to the localized laser wounding.

### Neurons and astrocytes respond differently to blast with shear

**Figure 4:**
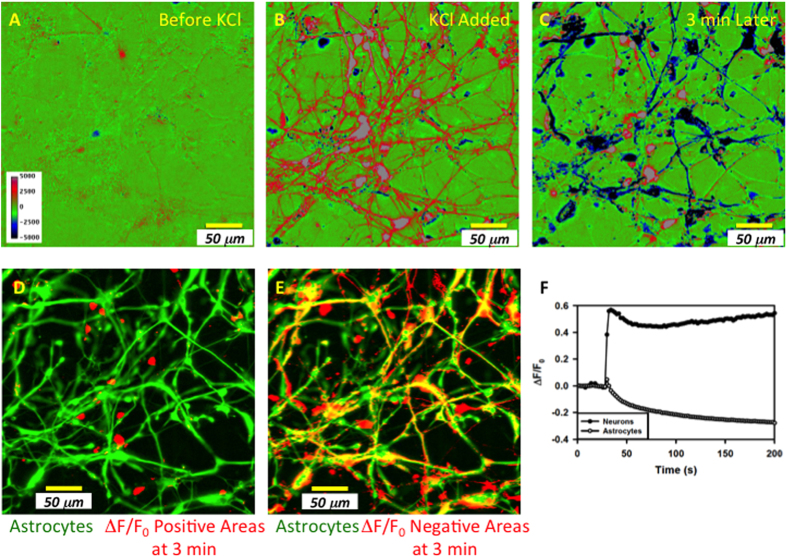
Astrocytes and neurons can be distinguished based upon their calcium response to potassium. Images A-C represent the calcium activity before (**A**), immediately after (**B**), and 3 minutes following the addition of KCl (**C**). The pseudo colors in images A-C represent the calcium activity around the mean activity observed prior to adding potassium. Positive activity (calcium increase above the mean) is in red/grey while negative activity (calcium decrease below the mean) is in blue/black. (**D**) Positive activity at 3 minutes (**C**) is represented in red and overlaid with the immunostaining for astrocytes, in green. No overlap between red and green is observed. (**E**) Negative activity at 3 minutes (**C**) now represented in red and overlaid with the immunostaining for astrocytes, in green; overlap, in yellow, is observed. (**F**) Average calcium activity in astrocytes and neurons using masks derived from (**C**) based on thresholds that separate the two populations. Scale bar, 50 μm.

Cells were identified as neurons or astrocytes using two different criteria: glial and neuron marker-specific immunostaining and/or the calcium response to KCl depolarization (see methods). Cellular ΔF Fluo-4 fluorescence before, immediately following, and 3 minutes after the addition of KCl in NB+B27 is shown in Fig. 4A,B and C. After 3 minutes, the ΔF activity separated into two classes, represented in the pseudo color image as red/grey (positive values) and blue/black (negative values) on a green background. The red/grey class did not co-localize with astrocyte immunostaining (Fig. 4D) while the blue/black class did co-localize with astrocyte immunostaining (Fig. 4E). Figure 4F shows the average calcium activity ΔF/F of the two cellular classes observed in Fig. 4C, now identified as neurons and astrocytes, using image masks derived from segmenting the two classes observed in Fig. 4C. The average number of astrocytes and neurons per experiment was 25 (4) and 28 (4), corresponding to ~47% and 53% of the cell population, respectively (mean (95% confidence), n = 43).

**Figure 5:**
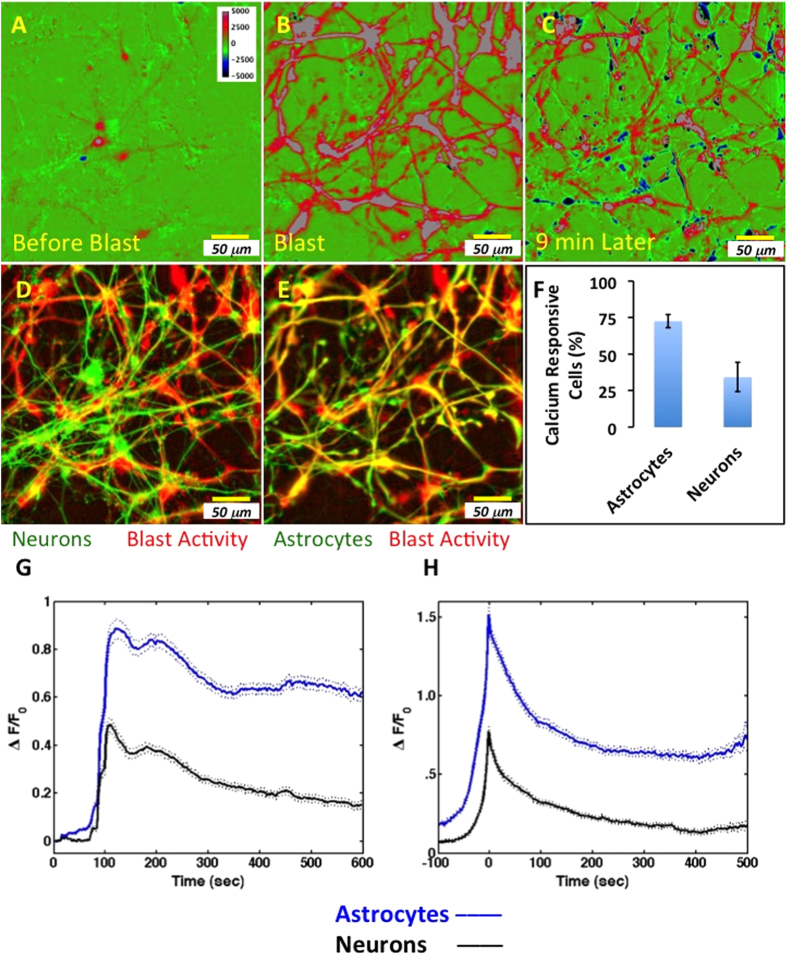
Calcium activity in response to blast occurs primarily in astrocytes. Images (**A**–**C**) represent the calcium activity before blast (**A**), after blast (**B**), and 9 minutes following blast. The pseudo colors (**A**–**C**) represent the calcium activity around the mean activity before the blast. (**D**) The activity represented in (**B**), in red, overlaid with the immunostaining for neurons, in green; note, minimal yellow consistent with minimal correspondence between activity and neurons. (**E**) The activity represented in (**B**), in red, overlaid with the immunostaining for astrocytes, in green; note, strong correspondence, indicated in yellow, between activity and astrocytes. (**F**) Percentage of calcium responsive astrocytes and neurons from their respective populations for all control blasts (mean +/− 95% confidence). (**G**) Average calcium activity, over all cells from 4 tissues, in astrocytes and neurons using masks derived from potassium challenge (mean, solid +/− 95% confidence, dotted, n = 1059 and 1173 astrocytes and neurons respectively, from 43 experiments). Simulated blast was triggered after ~100 seconds. (**H**) Peak centered average calcium activity in astrocytes and neurons of data presented in G. Scale bar, 50 μm.

To examine the extent that calcium responses to simulated blast are propagated by neurons or astrocytes, calcium levels were monitored continuously using the fluorescence signal ΔF before, during and after blast (Fig. 5A–C). The pseudo colors (Fig 5A–C) represent the calcium activity around the mean activity before the blast. The correlation between calcium activity and cell identity was first established by overlaying the activity image with the specific immunostaining for neurons and astrocytes, respectively (Fig. 5D,E). Cells that responded to the blast and cells that were identified as astrocytes by their immunostaining were spatially correlated (Fig. 5D vs. E). To quantify the correlation between blast response and cell type we evaluated first the percentage of responsive astrocytes and neurons from their respective populations identified using the KCl response. The fraction of calcium responsive cells varied in the two populations; 72% (5%) astrocytes and 34% (10%) neurons responded to blast (total population mean (95% confidence), n = 43 experiments from 4 independent human sources, consisting of 1059 and 1173 astrocytes and neurons respectively, Fig. 5F). The responding astrocyte fraction was significantly greater than the responding neuron fraction (p = 3.27 E−7 or p = 6.02 E−7, n = 43 for 2-tailed, paired t-test of direct fractions or Freeman-Tukey arcsin transformed fractions, respectively). To evaluate the response magnitude and time dependence in the responding populations, the ΔF(t)/F time course was determined using the KCl identified neurons and astrocytes (Fig. 5G). Within the respective responding populations, the astrocytic response was greater than the neuronal response. The blast response (integrated ΔF(t)/F time course) in the astrocytes was consistently and significantly greater than in the neurons (322.9 (62.1) and 110.2 (37.6), mean (95% confidence), for astrocytes and neurons, respectively; weighted averages over 4 tissues; p = 0.017, 2-tailed paired t-test). The neuronal response was ~30.6% (8.4%) (mean (95% confidence)) of the astrocyte calcium activity (Fig. 5G). To de-convolve the propagation dependent properties from the cellular response, a peak-centered average was determined (Fig. 5H); the magnitude and persistence of the astrocyte response was indicative of a larger calcium load compared to neurons. These differences were quantified by comparing the distribution of decay times and offset plateau values (time at which the peak ΔF(t)/F value decreased to 1/e and the fitted value of ΔF(t)/F at the end of the observation window). The distributions of decay times for astrocytes and neurons were significantly different (Kolmogorov-Smirnov, type 2, alpha = 0.02). The decay times were exponentially distributed (cumulative distribution function, CDF = 1 − exp(-(t-offset)/τ); n = 705 and 525 for astrocytes and neurons, respectively) with τ = 34.03 (1.85) and 10.78 (0.21) seconds (fitted τ (95% confidence)) for astrocytes and neurons, respectively, corresponding to a greater than 3 fold increase in the time during which calcium is elevated in astrocytes compared to neurons. The peak-centered average was fit as a single (astrocytes) or double (neurons) exponential with an offset; the offset value was significantly greater for astrocytes compared to neurons (0.62 (0.00) and 0.14 (0.00), offset (95% confidence)) indicating that the persistence of calcium was greater in astrocytes than in neurons after de-convolving propagation-dependent changes. In summary, both the proportion of responsive cells and the time-averaged responses to simulated blast with shear were significantly greater in astrocytes than in neurons.

### Calcium propagation in astrocytes occurs via purinergic signaling

**Figure 6:**
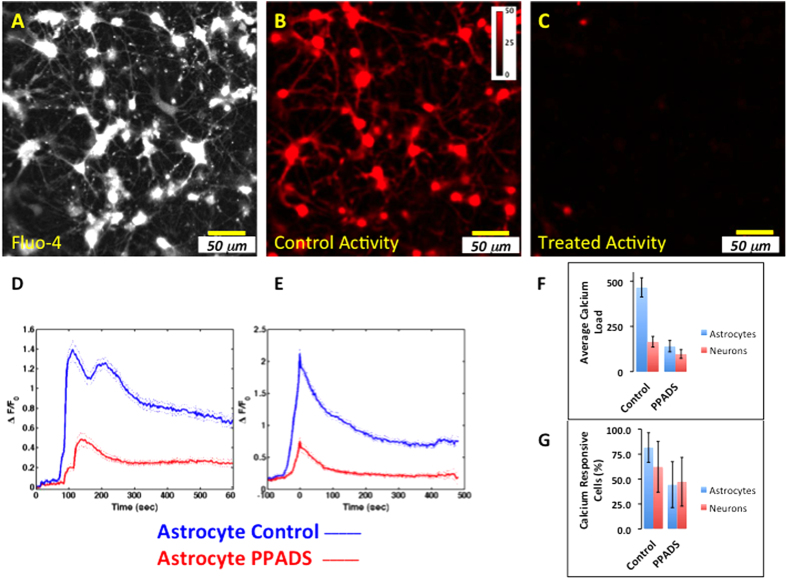
Calcium response to blast propagates via purinergic signaling. The same field of Fluo-4 labeled cells was exposed to blast in the presence and absence of PPADS. (**A**) Fluo-4 labeled cells. (**B**) Variance/Mean of the image sequence following the blast, control condition. (**C**) Variance/Mean of the image sequence following blast in the presence of PPADS. (**D**) Average calcium activity time course, in control and PPADS treated astrocytes using masks derived from KCl challenge (mean, solid +/− 95% confidence, dotted, n = 114, n = 7 matched experiments from 2 tissues). Simulated blast was triggered after ~100 seconds. (**E**) Peak centered average calcium activity time course in astrocytes and neurons of data presented in (**D**). (**F**) The calcium load (integrated response) in astrocytes is significantly decreased following PPADS treatment (140.98 +/− 31.23 vs. 465.17 +/− 52.82 mean +/− 95% confidence; p < 0.00001, 2-tailed unequal variance t-test). (**G**) The percentage of calcium responsive astrocytes was reduced significantly, to 44% +/− 23% (mean +/− 95% confidence) corresponding to an ~54% reduction in the number of responsive astrocytes while the fraction of calcium responsive neurons remained unchanged (n = 7, 8; p = 0.031 and 0.163, 1-tailed paired t-test, astrocytes and neurons, respectively. Scale bar, 50 μm.

We investigated whether the calcium response to simulated blast is dependent upon a purinergic signaling pathway as observed using laser wounding. The effect of purinergic ATP (P2) receptor inhibitors was evaluated in the same well and field of view following a control blast. Calcium levels were monitored continuously during the experiment; an example of the peak fluorescence signal observed following a control blast is shown (Fig. 6A). To represent the activity through time during and after a blast, the data were reduced to a single image (Fig. 6B) using the variance/mean calculated from the image sequence associated with Fig. 6A (see methods) over the time period ~10 sec prior to and ~150 sec following the blast. Following the first blast experiment, media was exchanged with equilibrated media containing 100 μM of the nonspecific purinergic antagonist PPADS, without moving the field of view, and a second blast was delivered after 10 minutes (Fig. 6C).

In seven of eight trials in which a first blast response was detected in the astrocytes, subsequently identified using KCl, the addition of 100 μM PPADS significantly reduced the blast response (Fig. 6D). To de-convolve the propagation dependent properties from the cellular response, a peak-centered average was determined (Fig. 6E). The integrated response of astrocytes treated with PPADS was significantly lower (140.98 (31.23) vs. 465.17 (52.82), mean (95% confidence); p < 0.00001, 2-tailed unequal variance t-test; Fig. 6F). Compared to control astrocytes, the treated response represents an ~70% (17%) (mean (95% confidence)) reduction in calcium activity. The integrated response of neurons treated with PPADS, while significantly lower compared to matched controls (97.82 (24.3) vs. 165.35 (29.53); p = 0.0002, 2-tailed unequal variance t-test) and with an ~41% (12%) (mean (95% confidence)) reduction in calcium activity represented a smaller change than that observed in astrocytes. PPADS treatment decreased both the magnitude and persistence of the response compared to match controls; both features are consistent with a decrease in the calcium load.

To further quantify these changes in astrocytes, the distribution of decay times, exponential offsets, and calcium responsive cell fractions were evaluated. In the presence of PPADS, the astrocyte decay times were exponentially distributed with τ parameter 8.26 (0.49) seconds (fitted τ (95% confidence)). This represents an ~20% reduction in the τ parameter compared to matched controls. The distributions of decay times for matched controls and PPADS treated cells were significantly different for both astrocytes and neurons (Kolmogorov-Smirnov, type 2, alpha = 0.01). The astrocyte exponential offset value was significantly lower following PPADS treatment (0.21 (0.00) vs. 0.68 (0.01), offset (95% confidence); PPADS vs. matched controls, respectively) indicating that the persistence of calcium was decreased in PPADS treated astrocytes after de-convolving propagation-dependent changes. The fraction of calcium responsive astrocytes was significantly reduced to 44% (23%) (mean (95% confidence)) corresponding to an ~54% reduction in the number of responsive astrocytes while the fraction of calcium responsive neurons remained unchanged (n = 7, 8; p = 0.031 and 0.163, using a 1-tailed paired t-test since the control fraction for astrocytes already approaches 1, astrocytes and neurons, respectively; Fig. 6G).

To control for a second blast effect, a second blast was given in the absence of PPADS; there were no significant decrease in either the fraction of calcium responsive cells (n = 5 experiments, p = 0.41 and p = 0.69, 2-tailed, paired t-test, astrocytes and neurons, respectively) or the time-averaged activity of astrocytes and neurons following a second blast (n = 5 experiments, p = 0.65 and 0.18, 2-tailed, paired t-test, for astrocytes and neurons, respectively). Blocking the ATP P2 receptors with PPADS significantly reduced both the proportion of responsive astrocytes and the time-averaged response of astrocytes to blast.

### Human and rat CNS cells respond differently to simulated blast

**Figure 7:**
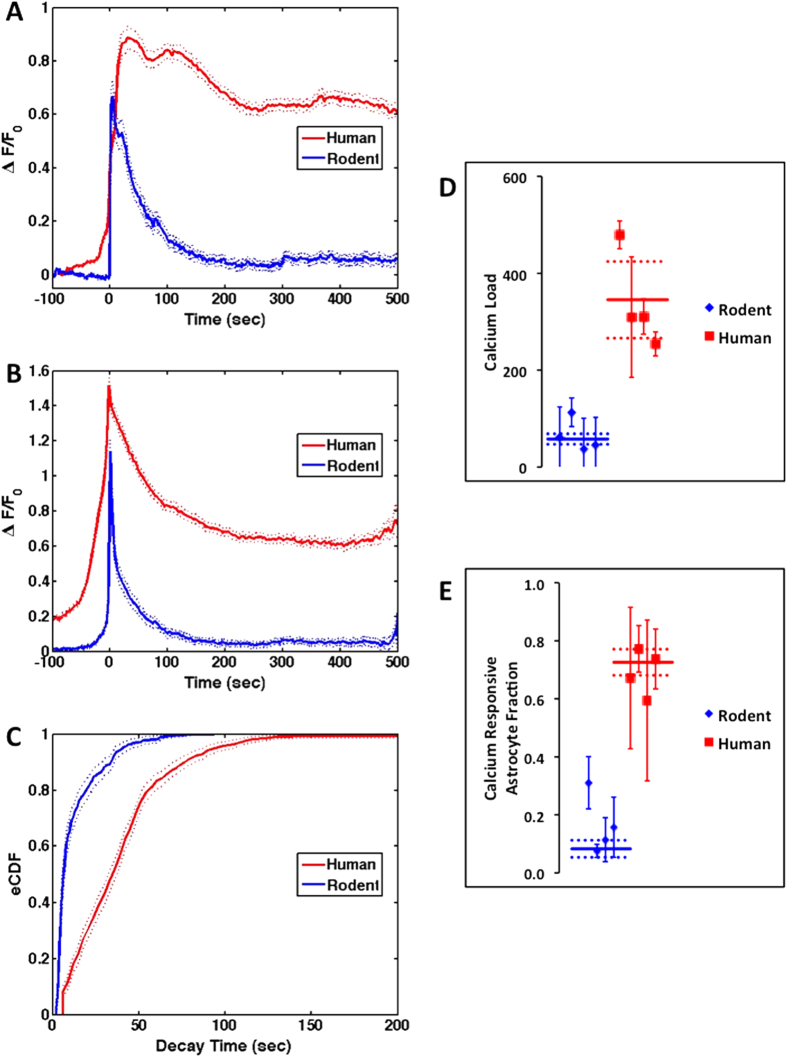
Human astrocytes have a prolonged calcium response compared to rat astrocytes. (**A**) Average calcium activity over all human and rat astrocytes responding to blast (red – human; blue – rat; mean, solid +/− 95% confidence, dotted, n = 4 and 4 independent tissues, 43 and 91 experiments and total astrocytes, 1059 and 1910, human and rat, respectively). (**B**) Peak centered average calcium activity highlights both the faster rise and decay times observed in rat and the response persistence observed in human. (**C**) The exponentially distributed response decay times (eCDF) are significantly longer in human astrocytes. (**D**) The calcium load (integrated response) in human astrocytes is greater across all 4 independent tissues compared to rat. Symbols represent weighted individual tissue means +/− 95% confidences while solid/dotted lines correspond to means +/− 95% confidences over all tissues. (**E**) The calcium responsive astrocyte fraction in human astrocytes is greater across all 4 independent tissues compared to rat. Symbols represent weighted tissue means +/− 95% confidences while solid/dotted lines correspond to means +/− 95% confidences over all tissues.

Since human astrocytes differ from rat we compared the responses of rat and human astrocytes to simulated blast. Rat astrocytes produce focal, transient and minimally propagating calcium responses (compare movies M2 to M1). Compared to rat, human astrocytes display a significantly larger calcium load (integrated ΔF(t)/F time course 322.9 (62.1) vs. 50.34 (12.20), mean (95% confidence), human vs. rat; Fig. 7A), a significantly longer time to peak and response persistence (time between half maximum and peak maximum ~12 vs. <2 sec and exponential offset value 0.62 (0.00) vs. 0.05 (0.00) (mean (95% confidence), human vs. rat; Fig. 7B), and a significantly longer distribution of decay times (Kolmogorov-Smirnov, type 2, alpha = 0.00001; Fig. 7C). The rat data summarizes the observations from 91 experiments and 1,910 astrocytes compared to the human from 43 experiments and 1059 astrocytes; both from 4 independent tissue sources (see methods). The rat response was independent of plating densities 12,500 to 60,000 cells/well (regression slope m not significantly different from 0; m = −0.47 (1.04); value (95% confidence)). The calcium response following blast decays slower and is incomplete (over 10 minutes) in human astrocytes (Fig. 7A,B). The rat decay times were exponentially distributed with τ parameter 10.16 (0.94) seconds compared to the significantly longer human τ parameter, 34.03 (1.85) seconds (fitted τ (95% confidence)). The calcium response in human astrocytes is greater across all 4 independent tissues compared to rat (Fig. 7D). The responding rat astrocyte fraction differed significantly from human: fractions 0.16 (0.10) and 0.69 (0.08) (mean (95% confidence) for rat and human respectively; Fig. 7E). For rat, the astrocyte fraction was independent of plating densities 12,500 to 60,000 cells/well (regression slope m not significantly different from 0; m = −0.72 (0.84); value (95% confidence)). Given the importance of intracellular calcium signaling in regulating cell function and gene expression, the larger and persistent calcium responses in human astrocytes following our simulated blast injury may suggest more potent *in vivo* effects in humans compared to rat.

### Simulated blast increases gene expression of GFAP and MMP-9 in human CNS cultures

**Figure 8:**
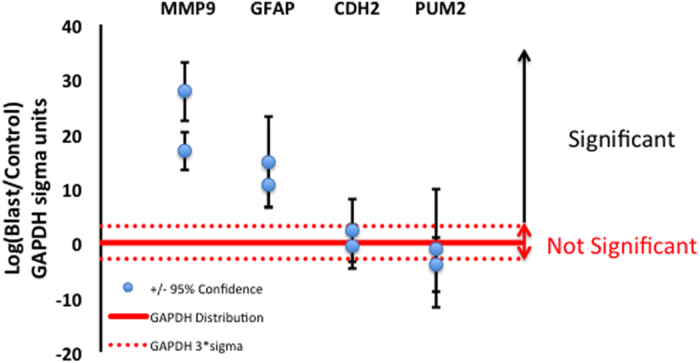
Blast-like shockwaves increase GFAP and MMP-9 expression in human CNS cells. Four genes related to TBI were evaluated 24 hours following blast stimulation; data from two independent experiments are shown. MMP-9 and GFAP were significantly elevated compared to control. Expression of CDH2 and PUM2 was not significantly different. Significance was evaluated using a 3 sigma threshold (99.7% probability; dotted red line) derived from analysis of the reference gene GAPDH (solid red line).

The chronic neurocognitive effects that can develop after blast in the absence of brain injury evidence indicate that a brief blast exposure may lead to persistent changes in cellular function. Here we report, for the first time, gene expression changes in human dissociated CNS cultures, 24 hours following conditions simulating mild bTBI (Fig. 8). Relative to control, MMP-9 and GFAP were elevated significantly under conditions shown to elevate astrocyte intracellular calcium concentration. Cadherin-2 (CDH2) and pumilio RNA-binding family member 2 (PUM2) expressions did not change significantly under our conditions. These four genes, MMP-9, GFAP, CDH2, and PUM2, are reported to alter their expression in rodents following impact and stab wound injury^18^,^19^,^20^ models that may be appropriate for the secondary and tertiary phases of bTBI. However, in a model of the primary phase of bTBI (blast tube), Affymetrix array analysis of rat hippocampus revealed mainly down-regulation of a number of gene families with no reported structural damage^21^. We observe that simulated blast comparable to blast conditions observed in mild bTBI can alter human gene expression of astrocyte-dependent pathways identified in TBI.

### Discussion

Utilizing a microscope system that allows exposure of human CNS cells to blast-like pressure profiles that are comparable to those associated with mild bTBI^7^,^8^, we found that in response to simulated blast a) astrocyte networks mediate propagating waves of elevated intracellular calcium, b) the calcium waves propagate via a purinergic signaling system and are blocked by P2 antagonists, c) human astrocytes respond with both a greater calcium load and an extended time course compared to rat astrocytes, and d) GFAP and MMP-9 mRNA expression in human CNS cells is increased after 24 hours. Pressure transients and shear forces used in this study were shown previously not to damage or effect human CNS cell survival^7^; an important consideration because minimal or no cell damage is observed in mild bTBI^22^.

In our system, the response of human CNS cells to simulated blast is the initiation of a propagating calcium response from one or more sites within the culture well. Astrocytes are the main carriers of this calcium wave. Astrocytes respond with higher proportion and calcium load compared to neurons; the calcium elevation occurs on the time scale of minutes. Blocking P2 receptors with the antagonist PPADS significantly reduced both the proportion of responsive astrocytes and the time-averaged calcium response of astrocytes without affecting the proportion of responsive neurons. The reduction in the time-averaged response of neurons was smaller than that of astrocytes. The calcium wave often propagates from outside the field of view, and can reach the field of view from different directions and distances; consequently, the apparent response time can differ between different culture wells. It is not clear what mechanism initiates the cellular response; focal membrane damage, activation of mechanoreceptors, and disruption of local cell contacts may all produce the initiating disturbance that results in the wave(s) of calcium activity^7^.

To determine if the blast-induced calcium response is the result of a common signaling pathway initiated from focal response sites we used laser injury to create a spatially defined injury site where calcium wave propagation was initiated. The response to laser induced focal damage was the generation of calcium activity that propagated throughout the cellular culture. This propagated calcium activity was blocked by apyrase-dependent ATP hydrolysis and by the P2 receptor antagonists PPADS; both observations are consistent with P2 receptor activation contributing to the propagated calcium activity. Furthermore, the propagated calcium activity was not dependent on neuronal communication since blocking neuronal activity with TTX, APV and BNQX had no effect. In both simulated blast and laser wounding, the calcium propagated response and the dependence on astrocytes and P2 receptors blockers were similar, consistent with the hypothesis that the calcium activity propagates via astrocyte-dependent pathways^12^. Inhibiting P2X7 receptors did not block the propagated calcium activity in laser wounded human astrocytes. This is interesting because P2X7 receptors are implicated in calcium signaling in rodent astrocytes^23^,^24^ and the response to trauma. For example, BBG rescues spinal cord injury in rats^25^.

The response to blast-like shock waves with concomitant shear forces differed between astrocytes from dissociated human CNS cortex, gestational weeks 19–21 and rat CNS cortex, embryonic day 18; human astrocytes have an increased calcium load and different propagation kinetics. These differences may result from species and/or developmental differences (human week 19–21 vs. rat E18, although reported for astrocytes to be developmentally comparable^26^), but not culture medium, incubator conditions, culture technique, cell densities, or assay techniques, which, in this study, were all the same for both rat and human. Human astrocytes do differ considerably from rodent astrocytes; both the astrocyte to neuron ratio^27^, and the complexity and size^27^ is higher in human astrocytes. Gene expression between different ages of human and rodents was shown to be different^26^. Human astrocytes maintain long cellular processes between the different layers of the cortex and the propagation of calcium waves is faster^28^. The more complex and elongated processes of the human astrocyte may be more sensitive to shear. The greater calcium load and prolonged kinetics observed in human astrocytes suggest that human astrocytes are more sensitive to blast with shear *in vitro*. The reasons for this difference may involve receptors, as there are known differences in receptors: the rat P2Y4 receptor responds to ATP as an agonist while in humans the same receptor responds to ATP as a competitive antagonist^29^.

The mild bTBI response to the primary blast phase can be relatively short, on the order of minutes to hours, but the pathophysiological consequences can last for much longer times. In our *in vitro* model of the primary blast phase, calcium elevations occurred primarily in astrocytes over a time scale of minutes without evidence for astrocyte damage or impaired survival^7^. If similar calcium waves occur *in vivo* as a response to the primary blast phase associated with mild bTBI, it is not clear what mechanistic cellular pathways are responsible for the long-term (hours to days or longer) effects observed in blast victims. In response to CNS injury, aberrant calcium elevations may be part of the pathological process. In rodent *in vitro* models, tertiary and secondary blast phases produce cellular responses involving astrocytes, including glial scar formation and formation of reactive astrocytes^19^,^30^,^31^,^32^. In injury models comparable to the secondary phase of blast, calcium elevation in response to injury induced increased production of GFAP, a marker for reactive gliosis, via a calcium-dependent N-cadherin up-regulation^19^. We suggest that in mild bTBI shear forces develop within the brain and these forces are dependent on brain-blast source orientation. The shear forces may create minimal focal damage and/or local activation of mechano-sensitive channels that results in propagated calcium activity mainly between astrocytes. Aberrant calcium elevations within a network of astrocytes can have many detrimental effects. For example, calcium elevation can cause vasoconstriction of blood vessels as a result of increased calcium concentration at astrocyte end feet^33^,^34^,^35^. Such vasoconstriction is observed in patients and in animal models of bTBI^36^,^37^. When astrocyte damage occurs *in vivo* as a result of a TBI, a number of pathways and mechanisms have been proposed to explain the loss of brain function including glutamate excitotoxicity due to impaired glutamate uptake^38^,^39^,^40^. Additionally, impaired uptake affects the availability of glutamine necessary for glutamate production^41^. Astrocyte involvement in TBI also can occur via energy demand and availability^42^. In response to TBI, alteration in ion-transporters can result in astrocyte swelling^43^. Astrocyte injury results in ATP release that activates purinergic receptors, elevates intracellular Ca^2+ ^44 and activates ERK^31^ and AKT pathways^44^ affecting gliosis, plasticity, and survival. The secretion of ATP and adenosine may stimulate secretion of trophic factors such as basic fibroblast growth factor (bFGF), nerve growth factor (NGF), ciliary neurotrophic factor, and S100β promoting neuroplasticity and nervous system recovery following injury^45^,^46^.

In our system, which models the primary blast phase with no evidence for injury and cell death during the first 24 hours, we found up-regulation of GFAP without up-regulation of the calcium-dependent N-cadherin pathway. This lack of change in the calcium-dependent N-cadherin pathway over 24 hours is consistent with the time course reported following stab-wound injury in which up-regulation was observed only 4 days after injury^19^. Apparently, the short calcium transients obtained in response to simulated blast are sufficient to increase GFAP expression, suggesting that multiple injury-dependent pathways influence GFAP expression and the resulting reactive astrogliosis. The up-regulation of GFAP, a marker of reactive astrocytes, can have long-term effects on the brain. MMP-9 expression was also up regulated; MMP-9 may play a crucial role in bTBI. One of the effects of MMP-9 is at the blood-brain barrier where barrier disruption could lead to long-term effects on brain function^47^. MMP-9 can also cause demyelination. In rat astrocytes the cytokine interleukin 1 beta (IL-1β) up-regulates the expression of MMP-9 through a Ca^2+^-dependent activation of Ca^2+^/calmodulin-dependent protein kinase type II (CaMKII) and Jun N-terminal kinase (JNK)^48^. We observed that MMP-9 mRNA levels, coding for a protein released by astrocytes in response to TBI, are elevated in our system within 24 hours following blast-like shock wave with shear.

Previous *in vitro* bTBI research primarily concentrated on injuries relevant to the secondary and tertiary (projectiles, impact) phases of blast injury^19^,^30^,^31^,^32^; phases that are both comparable to civilian TBI resulting from trauma and easier to simulate under *in vitro* conditions. Using a model system that replicates the physical properties of an explosive blast known to produce mild bTBI in humans, we have 1) determined a cell type (astrocytes) and pathway (purinergic) that propagates a calcium response in CNS cell cultures during the primary injury phase and 2) reported molecular changes (GFAP and MMP9 expression) that take place on a time scale much greater than the blast event. Since human astrocytes differ from rodent and other primate astrocytes structurally^49^, and physiologically^28^, it is not surprising that their responses to simulated blast also differ. Greater forces may be needed to replicate a comparable effect in the rodent, compared to human, CNS; this is an important consideration when model systems are used in the development of potential TBI treatments and countermeasures.

## Materials and Methods

### Ethical Approval

Primary, Sprague Dawley rat (Taconic, Germantown, NY) CNS cortex tissue was obtained from pregnant female rats euthanized by carbon dioxide asphyxiation; all procedures were carried out in accordance with the NIH guidelines for care and use of animals for experimental procedures approved by the NINDS Animal Use and Care Committee. Primary, human CNS tissue, gestational weeks 19–21, was obtained under surgical written informed consent in accordance to National Institutes of Health Institutional Review Board Exempt # 5116 under Johns Hopkins University approved protocols, based on its designation as pathological waste.

### Cell Culture

Cultures from primary, Sprague Dawley rat CNS cortex tissue, embryonic day 18, (E18) were prepared as described previously^50^. Since the plating survival density may be different between human and rat, rat cells were plated at different densities to evaluate whether changes in confluence contributed to the observed responses. Cells were plated at densities of 12,500, 25,000, 40,000, 50,000 and 60,000 per well (n = 18, 20, 16, 14, and 23, wells respectively) in NB + B27 in optical coverslip bottomed 96 well plates (Greiner, Monroe NC) that were threaded using a 1/16 NPT tap. The 96 well plates were coated with PureCol (Advanced BioMatrix, San Diego, CA; 6 mg/ml). The PureCol coated plates were then coated with poly-D-lysine (Sigma-Aldrich, St. Louis MO; 10 mg/ml) as previously described^7^. Half of the media volume was changed twice a week. Cells cultured for 2 to 4 weeks were used in all experiments. Cell preparations from cortices obtained from 4 pregnant rats, consisting of n = 46, 6, 18, and 21 individual experiments (91 total) were used in this study; the number of replicates for each experiment is indicated in the text.

Primary, human cortex CNS tissue, gestational weeks 19–21 was dissociated using gentle titration in Hank’s Basic Salt Solution (HBSS), centrifuged, washed and re-suspended in neurobasal medium (NB) supplemented with B27 (Invitrogen, Grand Island NY). Cells were plated at either a density of 50,000 cells/well in NB + B27 in optical coverslip bottomed 96 well plates (Greiner, Monroe NC) that were threaded using a 1/16 NPT tap or a density of 300,000 per 35 mm culture dish (MatTek, Ashland MA) for laser wounding experiments. The 96 well plates and 35 mm culture dishes were coated with PureCol (Advanced BioMatrix, San Diego, CA; 6 mg/ml). The PureCol coated plates were then coated with poly-D-lysine (Sigma-Aldrich, St. Louis MO; 10 mg/ml) as previously described^7^. Half of the media volume was changed twice a week. Cells cultured for 2 to 5 weeks were used in all experiments. For the blast studies, data from 4 independent human tissue sources, consisting of n = 7, 26, 5, and 5 individual experiments (43 total), are presented. For the laser wounding studies, data from 3 independent human tissue sources consisting of n = 8, 13, and 23 individual experiments (44 total) are presented. Each experiment involved an individual well and the number of replicates for each experimental condition is indicated in the text. The number of cells per single (non-tiled) field of view was comparable to our previous study^7^.

### Imaging System

The real time imaging system is a Ti wide-field inverted microscope with Perfect Focus (Nikon Instruments, Inc., Linthicum MD) equipped with an EM camera (Andor Technology, South Windsor CT; DU-897E) running NIS Elements (Nikon Instruments, Inc.) for data acquisition and experimental control. A 20x air objective (numerical aperture (NA) 0.75) and 60x oil immersion objective (NA 1.49) were used for imaging; 2x2 area stitching was used with the 60x objective. The fluorescence indicator, Fluo-4 (Invitrogen, Grand Island NY) was used to monitor intracellular calcium. Before each experiment, cells were loaded in the incubator for 30 minutes with 6 μM Fluo-4 AM in NB+B27 and maintained at 37 °C with 95% air and 5% CO_2_. At the end of the loading period, the cells were washed twice with NB+B27. An additional wash to the final well volume was done prior to imaging each well. Fluo-4 was excited using 480 nm light (Chroma Technology Corp., Bellows Falls VT; HQ 480/40 nm filter) and fluorescence emission collected (Chroma Technology Corp.; HQ 535/50 nm filter) during an exposure time of 80–100 msec. The image acquisition rate was 1.0–0.3 Hz. An external isolation box (Oko Lab, Italy) maintained temperature (37 °C) and blocked ambient light. A 96 well plate was mounted on the microscope stage using a custom clamp. To connect the pneumatic device to the 96 well plates, the microscope condenser was removed.

### Laser Wounding

When propagation of calcium activity from a defined initiation site was needed, laser wounding was used to injure cells and localize the initiating injury site to within the observation field in this same CNS culture system. A Zeiss LSM 410 laser scanning confocal system, converted to a 2-photon system (LSM Tech., Etters PA), with a 40x oil immersion objective (NA 1.3) was used for laser wounding studies. An ultra-fast laser (Verdi-V10/Mira-900; Coherent, Santa Clara CA) tuned to 800 nm produced ~1.8 W prior to entering the microscope. An external isolation box (Precision Plastics, Inc., Beltsville MD) maintained temperature (37 ^o^C) and blocked ambient light. Cells were maintained in a small environmental box (Oko Lab) supplied with a gas mixture of 95% air and 5% CO_2_. Cells were targeted and wounded using the fixed point-scan application in the LSM software for 5 seconds. The wounding power varied between 10–33% of the maximum laser power available at the sample. Wounding required that the laser be focused on the cell membrane because 5 μm deviations above or to the side were without observable effect. The wounding occurred at an energy threshold, required direct laser damage to the cell membrane, and was not the result of cavitation or indirect damage. Laser wounding creates a single injury site in which calcium activity propagates with a pattern of activity that was stereotypic and reproducible.

### Simulated Blast System

Although the system and methodology for exposing CNS cells to blast-like shockwaves while under simultaneous and long-term microscopic real time imaging has been described previously^7^,^8^, its design will be summarized here. The system incorporates a pneumatic device that is based on an air gun (AirForce Model R9901; Fort Worth, TX) modified by Axiom/Lemak (Linden, VA). The pneumatic device is attached to one of 96 wells positioned on a microscope and the amplitude of the pressure transient is varied with an adjustable quick release plug. We precisely and reproducibly control the properties of the gas released (composition, speed, volume, and timing); the gas mixture used was 95% air and 5% CO_2_, pressurized to 1500 psi. When activated, the pneumatic device simulates an open field explosive blast in a well of a 96 well plate. Previously, we showed that the simulated blasts were generated with ~0.1 msec rise times and a two component falling phase; the pressure waveform profile resembles the classic Friedlander curve with a fast component dropping below ambient pressure within 0.5 msec, and a slower component returning to ambient pressure within 2 msec. We identified that the response to blast occurred only in the presence of shear forces; all the experiments reported in this study were done in the presence of shear forces using a well volume of 110–150 μl and a peak pressure of ~6 atm that, according to our previous measurements, produce shear forces ≤1Pa^7^.

### Calcium Imaging

Prior to initiating simulated blast experiments, the covers of the 96 well plates were replaced by a gas tight film (EXCEL Scientific, Victorville CA; TSS-RTQ-100) sealing the wells of the plate, and preserving the gas environment inside each well^7^. The 96 well plate was attached securely to the motorized stage of the microscope. For each experiment, a single well was centered in the microscope’s field of view. The piece of film covering that well was cut and removed without affecting the sealing of the other wells, and a third wash established final well volume. The T-connector was then screwed into the well using the previously tapped threads. The high-pressure tubing was attached to the T-connector using a quick release connection. A quick release valve was secured in the T-connector with the other end of the high-pressure hose attached to the pneumatic device^7^. Baseline images were collected every 1–3 seconds for ~100 seconds; simulated blast was triggered after ~100 seconds while continuously imaging the well for 10 minutes. In laser wounding experiments, 2-photon excitation, Fluo-4 image sequences were collected with 1% of the available laser power at 800 nm at 0.2 Hz using the non-descanned detection mode. Calcium signaling was evaluated from the entire field of view or regions of interest by averaging fluorescence, ΔF/F as a function of time and then integrating the area under the ΔF/F curve for each experiment.

### Immunocytochemistry and Cell Identification

Cells were identified using two procedures: immunocytochemistry and their calcium response to KCl depolarization. For immunocytochemistry, cells were fixed with 4% paraformaldehyde and then stained for lineage-specific antigens without removing the live-cell chamber from the microscope stage. Cells were blocked in 5% normal goat serum with 0.1% Triton X-100, then labeled with two neuron-specific markers, class III beta-tubulin, TUJ1, (R&D Systems, Minneapolis MN; monoclonal antibody MAB1195) and microtubule associated protein 2, MAP2-Neuronal marker (clone AP-20 monoclonal antibody, A11268; Abcam, Cambridge MA), and a glia marker anti-Glial Fibrillary Acidic astrocyte marker, GFAP (polyclonal antibody Z0334; DakoCytomation, Denmark), and the nuclei marker, Hoechst (33343; Invitrogen, Grand Island, NY). GFAP is known to be a cell specific astrocyte marker applicable for human fetal astrocytes^51^. Secondary antibodies Alexa Fluor 594, goat-anti mouse IgG (H+L) and Alexa Fluor 488, goat anti-rabbit IgG (H+L) were purchased from Invitrogen, Grand Island NY.

For the calcium response to depolarization an equal volume of NB+B27+200 mM KCl was added to the well resulting in an excess 100 mM KCl in NB+B27. The rationale for using KCl depolarization to identify neurons and astrocytes was the following. In a single experimental session, up to 10 individual wells (experiments) from a single 96 well plate were used. For immuno-cytological identification each well was fixed following that experiment; at the conclusion of the entire series of experiments immunostaining was performed and, for each well, the identical field of view needed to be identified and reimaged. This was a very elaborate and time-consuming process and there were cases in which it was difficult to recover the identical area of interest. On the other hand, identifying cell type with KCl was rapid and robust and only required adding KCl at the conclusion of the individual experiment prior to investigating the next well.

### Image Analysis

Fluo-4 image sequences and immunofluorescence images, when acquired, were registered together using a rigid body transformation implemented in the ImageJ (NIH) plugin StackReg. To identify cells that responded with changes in Fluo-4 fluorescence to either a blast or KCl depolarization, the variance/mean intensity of a relevant time window from the image sequence before, during, and after the blast was created and, for KCl depolarization, a five frame average prior to KCl exposure was subtracted from each image (ΔF = F(t) − <F(t)>_Frames1−5_). The variance/mean image captures the changes over time in a single image and quantifies the calcium activity observed; this derived image was used as an aid in identifying calcium-responsive cells. Cell identity was determined at the end of the blast experiment using immunostaining for neuronal and astrocytic markers, and/or calcium response to KCl depolarization. Single cell analysis was used to quantify and identify cell types correlated with calcium activity triggered by simulated blast. Initially, regions of interest derived from the correlation between marker specific cell type (immunofluorescence) and the responses to depolarization were used to create identifying masks that were superimposed over all cells in the blast image sequence. Subsequent analysis, using only KCl depolarization, made use of ImageJ macros that automated the tasks of alignment, thresholding at 0.85 of the intensity cumulative distribution, object (region of interest; (ROI)) identification, image mask creation, and time series analysis. Time series data were then analyzed in Matlab using programs that allowed rapid operator data curation into responding and non-responding data sets for analysis and display. Since two methods of analysis were used, manual analysis and automated detection using 2 sigma threshold levels, all automated ROI identifications were validated manually and adjusted, if needed. All kinetic data were validated against the non-normalized image sequence; aberrant data, usually due to moving debris, were dropped.

### Gene Expression

In order to evaluate changes in gene expression following exposure to simulated blast, two primary human CNS tissue samples were dissociated and plated in two 96 well plates. Cells were grown as described in the cell culture method section for 21 days; simulated blast was delivered as described previously. As control, all steps in the procedure were repeated excluding the simulated blast itself. Blast-treated and control experiments were alternated on a row-by-row basis and then the 96 well plate was returned to the incubator. Samples remained in the incubator for an additional 24 hours at 37 ^o^C. Prior to RNA extraction, the wells were visually evaluated in the microscope in order to assess culture integrity; wells that showed cellular damage were excluded from analysis (n = 3, and n = 2, excluded blast and control wells from 60 wells for each tissue sample, respectively). RNA was collected using TRIzol reagents (Life Technologies) and then purified using RNeasy Mini Kit (Qiagen). The concentrations of RNA and 260/280 ratios were determined using a NanoDrop 2000 (Thermo Scientific). Equal amounts of RNA were used to make cDNA using SuperScript III First-Strand Synthesis System for RT-PCR (Invitrogen). Oligonucleotide sequences specific for genes of interest were designed with the Vector NTI Advance 11.5 program (Invitrogen). qRT-PCR determinations were performed in triplicate on an Applied Biosystems 7500 RT-PCR system with AB Power SYBG 2x master mix. Glyceraldehyde 3-phosphate dehydrogenase (GAPDH) expression was used as the reference for qRT-PCR analysis. Changes in the genes of interest were evaluated against variations in GAPDH detected in all samples. Calibration lines were determined for each probe using robust (bisquare) linear regression of threshold cycle vs. log amount. The weighted mean and weighted standard deviation of the reference probe GAPDH were determined from all measurements (4 independent samples each in triplicate). Rather than direct reference probe normalization (division by the paired GAPDH values), and the problems created by ignoring the reference probe distribution^52^, a distribution of log ratios was created by generating two sets of 10,000 normally distributed random samples using the GAPDH mean and standard deviation. The log ratio distribution, centered at zero (log_10_(1)) with standard deviation, sigma, represents the expected variation of the reference probe; changes greater than 3 sigma in the paired test probes were considered significant increases compared to the reference probe. This approach is similar to previous methods that try to incorporate the statistical properties of the reference probe^53^,^54^,^55^.

### Statistics

Significance between data sets was determined using ANOVA, t-test, or Kolmogorov-Smirnov and indicated in the text. The fraction of calcium responsive cells was evaluated after correcting for 0 and 1 fractions using Freeman-Tukey arcsin transformed, cell frequency weighted, pooled and back transformed averages; 0 and 1 fractions were assigned a value of 1/(4*n) and (1−1/(4*n)) where n is the total number of cells. Summary statistics were calculated over individual tissue averages using 1/variance weighting. 95% confidence intervals for the means were calculated using t*SEM where t is the two-tailed, 0.05 probability t-value and SEM is the standard error of the mean. Summary statistics, data analysis, and curve fitting were performed using the programs Excel (Microsoft, Redmond WA (summary statistics) and Matlab (Mathworks, Natick, MA (analysis and curve fitting)).

## Additional Information

**How to cite this article**: Ravin, R. *et al*. Blast shockwaves propagate Ca^2+^ activity via purinergic astrocyte networks in human central nervous system cells. *Sci. Rep*. **6**, 25713; doi: 10.1038/srep25713 (2016).

## Supplementary Material

Supplementary Information

Supplementary Movie S1

Supplementary Movie S2
