# Supplementary material for: Blast shockwaves propagate Ca2+ activity via purinergic astrocyte networks in human central nervous system cells
Source: Sci Rep. 2016 May 10;6:25713. doi: 10.1038/srep25713 (PMC4861979; doi:10.1038/srep25713)
Supplement: Supplementary Information [file srep25713-s1.pdf]

Blast shockwaves propagate Ca^{+2} activity via purinergic astrocyte networks in human central nervous system cells

Authors: Rea Ravin[1,2], Paul S. Blank[1], Brad Busse[1], Nitay Ravin[1,2], Shaleen Vira[1], Ludmila Bezrukov[1], Hang Waters[1], Hugo Guerrero-Cazares[3], Alfredo Quinones-Hinojosa[3], Philip R. Lee[4], R. Douglas Fields[4], Sergey M. Bezrukov[5], and Joshua Zimmerberg[1*]

1. Section on Integrative Biophysics, *Eunice Kennedy Shriver* National Institute of Child Health and Human Development, National Institutes of Health, Bethesda, MD 20892-1855
2. Celoptics Inc., Rockville, MD 20852
3. Department of Neurosurgery, Johns Hopkins University, Baltimore, MD 21287
4. Section on Nervous System Development and Plasticity, *Eunice Kennedy Shriver* National Institute of Child Health and Human Development, National Institutes of Health, Bethesda, MD 20892-3713
5. Section on Molecular Transport, *Eunice Kennedy Shriver* National Institute of Child Health and Human Development, National Institutes of Health, Bethesda, MD 20892-0924

* Corresponding Author:
Dr. Joshua Zimmerberg
Eunice Kennedy Shriver NICHD, NIH
Building 10, Room 10D14
Bethesda, MD 20892-1855
zimmerbj@mail.nih.gov
301-496-6571
Fax: 301-480-2412 or 301-480-0857

M1. Calcium propagated response to blast shock wave with shear in Fluo-4 labeled human CNS culture. The acquisition rate was 0.33 Hz; each image represents a 2x2 stitching of adjacent areas with 15% overlap between areas.

M2. Calcium propagated response to blast shock wave with shear in Fluo-4 labeled rat cortex CNS culture. The acquisition rate was 1.0 Hz.
